# International Society for Quality of Life Research commentary on the US Food and Drug Administration draft guidance for industry on core patient-reported outcomes in cancer clinical trials

**DOI:** 10.1007/s11136-023-03396-z

**Published:** 2023-05-23

**Authors:** Keri J. S. Brady, John Devin Peipert, Thomas M. Atkinson, Cecilia Pompili, Monica Pinto, James W. Shaw, Jessica Roydhouse, The International Society for Quality of Life Research Clinical Practice Special Interest Group, the International Society for Quality of Life Research Clinical Practice Special Interest Group, the International Society for Quality of Life Research Regulatory Health, Technology Assessment Engagement Special Interest Group, and the International Society for Quality of Life Research Psychometrics Special Interest Group

**Affiliations:** 1 Sanofi, Cambridge, MA USA; 2 Department of Medical Social Sciences, Northwestern University Feinberg School of Medicine, Chicago, IL USA; 3 Memorial Sloan Kettering Cancer Center, New York, NY USA; 4 Patient-Centred Outcomes Research (PCOR), University of Leeds, Leeds, UK; 5 Rehabilitation Medicine Unit, Istituto Nazionale Tumori – IRCCS – Fondazione G. Pascale, Naples, Italy; 6 Bristol Myers Squibb, Lawrenceville, NJ USA; 7 Menzies Institute for Medical Research, University of Tasmania, Hobart, TAS Australia

**Keywords:** Patient-reported outcomes, Clinical outcome assessment, Guidance, Oncology, Trial

## Abstract

In June 2021, the US Food and Drug Administration (FDA) released a draft guidance for industry on core patient-reported outcomes (PROs) and related considerations for instrument selection and trial design in registrational cancer clinical trials, building on prior communications about the use of PROs to assess efficacy and tolerability in oncology drug development. The International Society for Quality of Life Research (ISOQOL) Standards and Best Practices Committee led an initiative to draft a commentary about the guidance, focusing on its positive aspects and areas that would benefit from additional clarification and consideration. For comprehensiveness, the authors reviewed existing public comments on the draft guidance, and the commentary underwent a thorough review process through three ISOQOL Special Interest Groups (Psychometrics, Clinical Practice, and Regulatory and Health Technology Assessment Engagement) followed by the ISOQOL Board. The goal of this commentary is to situate this new and relevant guidance document within the context of recent regulatory efforts on PROs and highlight areas in which further work may ultimately benefit the field.

## Background

Patient-reported outcomes (PROs), defined as “any report of the status of a patient’s health condition that comes directly from the patient, without interpretation of the patient’s response by clinicians or anyone else” [1, p. 2], are considered to be the gold standard representation of the patient experience [2, 3] and are commonly included in randomized clinical trials in oncology [4]. Since 2009, regulatory agencies such as the United States Food and Drug Administration (FDA) and European Medicines Agency (EMA) have released guidance [1, 5] and reflection papers [6] regarding the use of PROs in drug development. There have also been publications relating to efforts by regulatory bodies to consider PRO-related topics in oncology. For example, FDA authors published a 2016 paper discussing core PROs in cancer trials [7], and a summary of a FDA/Critical Path Institute workshop about the use of PROs to inform the assessment of tolerability in cancer trials was published in 2018 [8]. In 2021, FDA released a draft guidance for industry on core PROs in cancer clinical trials (hereafter referred to as *FDA Core PRO in Cancer Trials Draft Guidance*) [9], and the public had the opportunity to comment. The FDA Core PRO in Cancer Trials Draft Guidance provides recommendations to sponsors on the collection of a core set of PROs and related considerations for instrument selection and trial design in registrational trials of anti-cancer therapies intending to demonstrate an effect on survival, tumor response, or delay in disease progression [9]. The guidance discusses disease-related symptoms, symptomatic adverse events, overall side effect impact summary measures, physical function, and role function as core PROs [9].

Regulatory guidance and commentary can provide valuable information on regulatory thinking and will likely influence choices and considerations by sponsors and researchers regarding the use of PROs in cancer trials. Thus, the International Society for Quality of Life Research (ISOQOL) Standards and Best Practices Committee sought to organize a broader comment on the FDA Core PRO in Cancer Trials Draft Guidance by groups within ISOQOL. The Standards and Best Practices Committee writing group included members from industry, academia, and clinical practice with expertise in oncology, psychometrics, clinical outcome assessment, and trial design. ISOQOL members were recruited by an email call put through the Clinical Practice Special Interest Group (SIG) and Psychometrics SIG. Patients were not involved in the writing team. The writing team also reviewed the public comments on the FDA Core PRO in Cancer Trials Draft Guidance [10]; a total of 28 were listed on the webpage, and 25 were relevant (3 were duplicates and/or spam). To ensure comprehensive review and feedback, the draft commentary was circulated to the Clinical Practice, Psychometrics, and the Regulatory and Health Technology Assessment Engagement SIGs prior to review by the ISOQOL Board. The goal of this commentary is to provide a perspective on the FDA Core PROs in Cancer Trials Draft Guidance with particular focus on highlighting its positive aspects as well as areas in which additional consideration may be beneficial or challenges for the field remain. As regulatory guidance can have a broad impact on the field, the intended audience for this perspective includes anticipated end-users of the guidance, including PRO researchers and trialists in regulatory agencies, industry, and academia.

## Commitment to patient voice by establishing core PROs as a minimum expectation

Critically, the FDA Core PROs in Cancer Trials Draft Guidance demonstrates FDA’s continued commitment to patient-focused drug development and provides a strong signal to sponsors, researchers, and trialists that the inclusion of PROs to assess efficacy and tolerability in registrational oncology trials is a clear expectation. This commitment was positively viewed in many public comments. Although prior work published by FDA authors has proposed a similar set of core PROs for oncology trials [3], such publications are not equivalent to guidance. The current FDA Core PROs in Cancer Trials Draft Guidance expand upon previously published core PROs, offering sponsors and trialists a foundational framework for defining *which* PROs should be assessed in registrational trials of anti-cancer therapies with important considerations for *how* to assess them. Importantly, the guidance provides sponsors and trialists the flexibility to determine this, given these decisions require stakeholder (including patient) input and consideration of the context of use.

The core PROs proposed in the FDA Core PROs in Cancer Trials Draft Guidance represent a ‘minimum expectation’ for PRO data collected in registrational oncology trials. The concept of a ‘minimum expectation’ for PRO data in cancer clinical trials, as described in this draft guidance, is an important step since it should improve the comparability of PRO data across trials. For this reason, the ISOQOL commentary on the 2014 EMA reflection paper had already noted the usefulness of a core set of PROs [11]. Furthermore, the FDA Core PROs in Cancer Trials Draft Guidance are well aligned with other ongoing PRO standardization efforts in oncology such as the Setting International Standards in Analysing Patient-Reported Outcomes and Quality of Life Endpoints Data Consortium (SISAQOL) [12]. SISAQOL’s initial work on analysis has subsequently been expanded on by the SISAQOL-IMI consortium [13].

However, it is important to highlight that many cancer trials may require additional PRO or other patient-reported data not captured by the minimum core set, depending on trial and stakeholder needs. In these cases, the minimum core set should be a starting consideration for the trial design team. A potential risk of identifying a minimum core set is the possibility that this set is interpreted as sufficient coverage of all important elements of the patient experience during therapy without due consideration of the specific needs of a trial. Because it is critical to ensure that PRO data are used if they are collected, a multi-stakeholder discussion is needed on how PRO data outside the core minimum dataset can inform the understanding of therapeutic risk and benefit for different therapies as well as be communicated more broadly, as suggested by public comments and the guidance itself. This discussion could also help harmonization of guidance and recommendations pertaining to PROs that come from different regulatory bodies (e.g., FDA, EMA, HTA bodies, etc.). Such discussions may be particularly relevant for specific populations such as pediatric, adolescent, and young adult populations, as indicated by public comment.

## Use of core PROs to assess treatment tolerability

The FDA’s inclusion of core PROs to assess both the efficacy and tolerability of investigational anti-cancer therapies is valuable. Tolerability can be defined as the degree to which adverse effects associated with a therapy impact a patient’s ability or desire to adhere to the therapy and/or its dose and includes direct measurement from the patient on how they are feeling and functioning during treatment [14, 15]. The discussion of PROs for labeling claims in the 2009 FDA guidance [1] was more focused on efficacy endpoints. However, reliance on clinician assessment of symptomatic toxicities can lead to an underestimation of patients’ experiences while on therapy [16], and recognition of this has led to efforts such as the Patient-Reported Outcomes Version of the Common Terminology Criteria for Adverse Events (PRO-CTCAE) [17]. To date, explicit statements of whether specific PROs have been included for either efficacy or tolerability is rare in cancer trial protocols, as most protocols merely feature a single PRO-focused section that does not fully specify the purpose of the PROs in the trial. Likewise, it is rare for PROs to be mentioned in the safety and tolerability sections of trial protocols. Therefore, differentiating these two different potential uses of PROs in cancer trials may lead to additional clarity and refinement of PRO strategies in this setting.

While the FDA Core PROs in Cancer Trials Draft Guidance’s discussion of tolerability underscores the value of PRO data in this context, additional thinking and consideration regarding the use and operationalization of tolerability endpoints could enhance this further. In particular, clarification on how patient-reported tolerability assessments (i.e., symptomatic AE and overall side effect impact measures) will be used in regulatory decision-making, such as in the formal benefit-risk assessment, in labeling, or as supplemental/contextual data, is needed. Such clarification will better ensure that both trial designs and the evidence used to support tolerability endpoints are appropriate for their intended use. In addition, such clarification can inform patient expectations regarding data collection as well as discussions between patient and research stakeholders. In addition, although the FDA’s efforts to differentiate tolerability assessments using PROs from safety assessments provides important guidance for trial design, public comments suggested that a more explicit statement that PROs would not be included in safety assessments may be even more helpful. An earlier publication by FDA authors emphasized that clinical safety data (i.e., CTCAE), not PRO tolerability data, are used for safety reporting [18]. Furthermore, discussing how PRO tolerability data and standard CTCAE data can be used together to provide a comprehensive picture of a drug’s safety and tolerability is an important conversation for stakeholders going forward.

A related question in need of clarification and discussion among stakeholders (patients, sponsors, researchers, trialists, clinicians, health authorities) is if tolerability can be considered comparatively in addition to descriptively. This is especially relevant as anti-cancer treatments are increasingly aiming to differentiate on the basis of treatment tolerability. Importantly, this is an area in which additional methods development is required, and thus expecting regulatory guidance may be premature. In particular, development of methods for operationalizing comparative PRO tolerability endpoints for use in the benefit-risk assessment and labeling are needed. For example, the draft guidance highlights several single-item patient-reported tolerability measures, such as individual PRO-CTCAE items and overall side effect impact items (e.g., Functional Assessment of Cancer Therapy—Item GP5 [FACT-GP5] [19] or European Organisation for Research and Treatment of Cancer—Question 168 [EORTC Q168] [20]). However, if there is stakeholder interest in treatment comparisons based on tolerability, then methods for determining meaningful within-patient change thresholds for these single-item measures need to be discussed.

Another important question relating to tolerability that will require discussion involving multiple stakeholders is methods for selecting patient-reported symptomatic AEs in tolerability assessment. The FDA Core PRO Draft Guidance suggests selection of the “most important and/or high frequency AEs,” to minimize patient burden. Yet, the task of balancing the need for a comprehensive set of specific AE items to cover patients’ experience with toxicity and the need to reduce participant response burden is challenging. While there have been publications regarding patient-reported AE item selection [21, 22], in the absence of specific guidance, researchers may feel the need to use a lengthy list of symptomatic AE items to avoid missing important AE signals [23], especially when specific symptomatic AE profiles may differ substantially across treatment arms. In such situations, clarification and discussion is needed to minimize subjectivity in the selection of “important” AEs, which in turn will likely lead to bias. However, it is important to avoid missing important symptoms and AE signals [23]. Although questionnaire length should be considered, it alone should not be the deciding factor when evaluating tolerability as patients may be willing to complete longer measures [24, 25]. In addition, in these cases, overall side effect impact or the use of item libraries may be more optimal. Clarification and discussion across multiple stakeholders are needed to help achieve a balance between the unbiased assessment of symptomatic AEs and respondent burden.

Given the inclusion of both disease-related symptoms and symptomatic AEs as core PROs in the FDA Core PROs in Cancer Trials Draft Guidance, another consideration is how to achieve a minimally burdensome yet unbiased symptomatic AE assessment for symptoms that are both related to disease and treatment. Disentangling disease- versus treatment-related symptoms may be difficult or infeasible in some contexts (e.g., fatigue among non-small cell lung cancer or NSCLC patients [26]). Furthermore, measuring symptoms that are both disease- and treatment-related through both a specific disease symptom measure (e.g., Non-Small Cell Lung Cancer Symptom Assessment Questionnaire [NSCLC-SAQ] [27]) and through patient-reported AEs (e.g., using PRO-CTCAE) can result in duplication and unnecessary patient response burden. Clarification on PRO selection for tolerability assessment when symptomatic AEs are redundant with disease symptom items or scales in other questionnaires is needed. In addition, while there is clearly value in collecting free-text symptomatic AE data, it can be operationally challenging to implement this in large-scale pivotal trials (e.g., due to volume of data collected, need for translations, qualitative analysis, etc.). These concerns were also reflected in public comments. Free-text PRO data collection in early-phase trials may be more useful in informing symptomatic AE collection in later phase trials. In addition, free-text PRO data collection may also be useful in identifying unexpected AEs. Consideration of this issue as part of a broader discussion around PROs and tolerability in early-phase trials, including trials involving multimodality treatments such as surgery, may be informative.

## Inclusion of PROs in early-phase trials

An important consideration for stakeholders going forward is the need for methods development to support guidance on the use of PROs in early-phase trials. As noted above, many oncology trials tend to use accelerated designs, underscoring the question of when and how to include PROs in early-phase or accelerated trials. The issue of using PROs to support submission of therapies on accelerated approval pathways was raised in public comments. The relevance of PROs in dose optimization in oncology has been discussed for some time [28], and PRO use in phase I trials is infrequent but has been increasing [29, 30]. There has also been work in the statistical literature evaluating methods for including PRO data in early-phase trials [31]. More recently the FDA-American Society of Clinical Oncology (FDA-ASCO) virtual workshop on optimizing dose selection strategies in oncology highlighted a novel approach for using PRO-CTCAE data as a complement to traditional CTCAE data in exposure response analyses [32]. The Friends of Cancer Research (FOCR) also convened industry, academic, regulatory, and patient advocate representatives to identify opportunities, challenges, and solutions for collecting PROs in dose optimization studies [33]. However, the absence of regulatory guidance on best practices for designing, analyzing, and interpreting PRO endpoints in early-phase trials and how this information might be considered by key stakeholders is a challenge. This presents opportunities for collaborative PRO research involving patients, PRO researchers, trialists, statisticians, and clinicians to inform future regulatory guidance.

## Core PROs in labeling

Another helpful area of discussion in the FDA Core PRO Draft Guidance was regarding how exploratory PRO findings may be considered for labeling claims. ISOQOL’s 2014 commentary discussed the importance of regulatory guidance regarding PRO labeling claims, as this can help guide sponsors’ PRO strategy and ultimately trial design [11]. The discussion of how exploratory PRO data were used to further describe symptomatic AEs in the case of Xalkori (crizotinib), a targeted therapy for non-small cell lung cancer, provides insight into how descriptive PRO data can inform labels, and thus how PRO data can be used for understanding treatment risk and benefit. The FDA’s label for Xalkori discussed patient-reported visual symptoms experienced [34].

Although the section on labeling considerations provided helpful insights into how PRO data can be considered for labeling, additional information and clarification would be helpful given the relatively infrequent inclusion of PROs in FDA drug labels. A review of breast cancer therapies approved by FDA from 2000 to 2019 found that a majority of trials included PROs, which despite being discussed in FDA review documents were not included in drug labels [35]. Another study that looked at products approved by the FDA’s oncology office from 2010 to 2014 found that only three of 40 products had PROs in the label [36]. Differences between FDA and EMA inclusion of PROs in oncology drug labels have been reported previously [37]. The nature of oncology trial submissions may be a factor: compared to non-oncology submissions, oncology products tend to be submitted as priority, fast track, and accelerated review, and oncology trials tend to have challenging features such as single-arm designs [36]. Given this infrequent inclusion, more information on how PROs are being considered could help drive improvement. Statements regarding the importance of missing data and other challenges to trials are important, but information about the elements of good design can also be beneficial. The statements regarding the importance of pre-specification and multiplicity in the guidance are helpful in this regard. As reporting and communication of PRO findings from trials can help translate PRO results into impact [38], discussion of the value of PRO data and how PRO findings can be communicated would be useful for researchers, regulators, sponsors, and patients.

## Trial design considerations

With regard to data collection approaches, the FDA Core PROs in Cancer Trials Draft Guidance’s modular approach to assessment is an encouraging step towards reducing response burden in clinical trials. For example, existing instruments like the European Organisation for Research and Treatment of Cancer (EORTC) core Quality of Life Questionnaire (QLQ-C30) include multiple unidimensional subscales that are currently typically administered in full, providing scores on all subscales, whereas not all subscales may be affected by treatment at a given timepoint. A modular approach selecting relevant subscales or approaches such as building custom PRO measures using items from libraries are available [20, 39], which could allow for a greater focus on clinically relevant PRO domains. However, it is not clear if this proposed approach will be accepted by other stakeholders such as the EMA or health technology assessment (HTA) bodies. Requests in the public comment for more information about the modular approach suggest further discussion of such approaches can be beneficial. In the absence of harmonization across health authorities on this topic, sponsors will continue to use the standard approach as the least risky option. We, therefore, encourage additional guidance and information on issues such as this from other health authorities to clarify areas of alignment and non-alignment, thus, leading to PRO strategies that minimize respondent burden to the greatest extent possible.

In addition, although the modular approach is a positive step, the connection between the core minimum dataset and PRO collection in earlier phase trials would benefit from further discussion. If, for example, tolerability and efficacy hypotheses pertaining to the core PROs have been previously tested in early-phase trials, in some cases, it may be that only core PROs expected to show a clinically meaningful treatment effect should be included in a pivotal trial. Such clarification can help with making trials even more patient-centric and minimizing response burden. In addition, thinking from other regulatory bodies and stakeholders in terms of how such an approach could be harmonized would be helpful.

In addition, it is positive to see FDA consider the timing of assessments. The Standard Protocol Items: Recommendations for Interventional Trials—Patient-Reported Outcome (SPIRIT-PRO) Extension, which provides guidelines regarding the PRO content of trial protocols, includes a PRO assessment schedule as one of its checklist items [40]. The FDA has also recently published research demonstrating that different PRO assessment schedules can yield different interpretations of PRO results [41], further underscoring the importance of providing guidance on assessment time points. Timing can also be relevant for overlapping symptoms and AEs.

Furthermore, the FDA Core PRO in Cancer Trials Draft Guidance’s statement regarding the use of ePRO and assessments out of clinic to minimize patient burden is important. It would be further strengthened by providing more information regarding the evidentiary standards sponsors should meet to demonstrate measurement comparability of PRO data collected via different modes of administration (e.g., home- vs. site based). The sample assessment frequency in the FDA Core PRO in Cancer Trials Draft Guidance suggests weekly assessment in the earlier cycles of treatment. Weekly assessment of PROs in earlier cycles of treatment will most likely require home-based assessments, which may be followed by site-based assessments for the trial period in which PRO assessments are less frequent and aligned with treatment visits. In addition, oncology trials may involve multimodality treatments when surgical inpatient episodes are mixed with site-based assessments. Further discussion on regulatory expectations regarding the demonstration of mode invariance across home- versus site-based PRO assessments would be beneficial. The International Society for Pharmacoeconomics and Outcomes Research (ISPOR) has a Task Force on measurement comparability across modes [42], and the Task Force’s report can help guide trial design. Citation of the Task Force report in future guidance documents can be helpful in directing researchers to this resource. Relatedly, the COVID-19 pandemic accelerated interest in decentralized or virtual trials [43], and the FDA’s guidance on trials during the COVID-19 pandemic discussed considerations for remote PRO assessment [44]. In light of the strong interest in decentralized trials [43, 45, 46], greater discussion and consideration of issues such as mode invariance in a more virtual trial era can advance the field.

Lastly, resources to support best practices for PROs in trials that can help researchers and trialists are available on the Patient-Reported Outcomes Tools, Engaging Users, and Stakeholders (PROTEUS) website [47]. These include the Consolidated Standards of Reporting Trials—Patient-Reported Outcome Extension (CONSORT-PRO extension) [48], the SPIRIT-PRO extension [40], and SISAQOL [12]. Citing such references in guidance documents can be helpful in encouraging uptake of best practices. In addition, the International Council for Harmonisation of Technical Requirements for Pharmaceuticals for Human Use (ICH) recently released the estimand framework (ICH E9 [R1]) to better connect trial goals, design, and analysis [49]. The relevance of the estimand framework for PROs, including in oncology, has been discussed [50, 51]. Citation of this document was suggested in public comment. Finally, disparities in cancer clinical trial participation are well recognized [52], and there are regulatory efforts to enhance equity in cancer trials [53]. PRO data collection should not be exempt from such considerations and efforts [54].

## Considerations for future trial design from a PRO research perspective

In summary, the FDA Core PRO Draft Guidance is an exciting step forward for the field. The draft guidance underscores the importance, relevance, and value of PRO data in drug development. The discussion of PRO data for both tolerability and efficacy endpoints and the concept of a minimum dataset are important steps for driving future patient-centered cancer trials. Areas in which further clarification would be beneficial include discussion of communicating data from and about patients, including PRO data that go beyond the core outcomes. Other areas include the balance between patient burden and unbiased assessment of core and other PROs and the use and operationalization of tolerability endpoints. Further areas include the collection of PRO data in early-phase and accelerated trials, which are common in oncology and considerations regarding PRO data collection and mode invariance in decentralized trials. Addressing these areas can enhance the patient centricity of future oncology trials and optimize the use of valuable patient data.

## Data Availability

Not applicable.
